# Computational-Based Polyphenol Therapy for Nonsmall Cell Lung Cancer: Naringin Coamorphous Systems for Solubility and Bioavailability Enhancement

**DOI:** 10.1021/acs.molpharmaceut.4c00303

**Published:** 2024-07-25

**Authors:** Dani Lakshman Yarlagadda, Subham Das, Sai Krishna Anand Vullendula, Suman Manandhar, Swapnil J. Dengale, K. Sreedhara Ranganath Pai, Krishnamurthy Bhat

**Affiliations:** † Department of Pharmaceutical Quality Assurance, Manipal College of Pharmaceutical Sciences, Manipal Academy of Higher Education (MAHE), Manipal, Karnataka 576104, India; ‡ Department of Pharmaceutical Chemistry, Manipal College of Pharmaceutical Sciences, Manipal Academy of Higher Education (MAHE), Manipal, Karnataka 576104, India; § Department of Pharmacology, Manipal College of Pharmaceutical Sciences, Manipal Academy of Higher Education (MAHE), Manipal 576104, India; ∥ Department of Pharmaceutical Analysis, National Institute of Pharmaceutical Education and Research (NIPER), Guwahati, Changsari 781101, India

**Keywords:** computational simulations, lung cancer, coamorphous systems, polyphenols, ceritinib, naringin

## Abstract

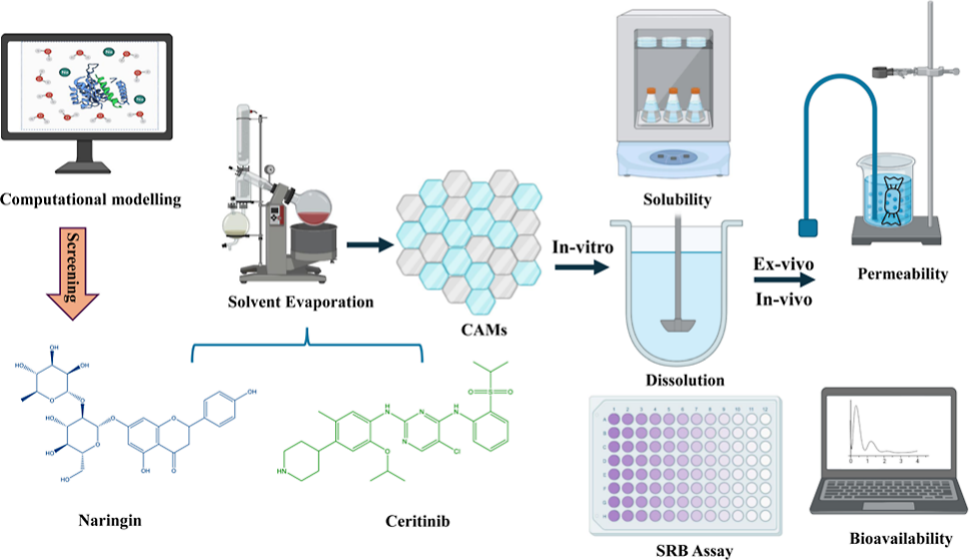

In this research, we utilized molecular simulations to create co-amorphous materials (CAMs) of ceritinib (CRT) with the objective of improving its solubility and bioavailability. We identified naringin (NRG) as a suitable co-former for CRT CAMs based on binding energy and intermolecular interactions through computational modeling. We used the solvent evaporation method to produce CAMs of CRT and NRG, expecting to enhance both solubility and bioavailability simultaneously. The solid-state characterization using techniques like differential scanning calorimeter, X-ray powder diffraction, and Fourier-transform infrared spectroscopy affirmed the formation of a single amorphous phase and the presence of intermolecular interactions between CRT and NRG in the CAMs. These materials remained physically stable for up to six months under dry conditions at 40 °C. Moreover, the CAMs demonstrated significant improvements in the solubility and dissolution of CRT (specifically in the ratio CRT:NRG 1:2). This, in turn, led to an increase in cytotoxicity, apoptotic cells, and G0/G1 phase inhibition in A549 cells compared to CRT alone. Furthermore, CRT permeability is also improved twofold, as estimated by the everted gut sac method. The enhanced solubility of CAMs also positively affected the pharmacokinetic parameters. When compared to the physical mixture, the CAMs of CRT:NRG 2:1 exhibited a 2.1-fold increase in CRT exposure (AUC_0–*t*_) and a 2.4-fold increase in plasma concentration (*C*_max_).

## 1 Introduction

Lung cancer is one of the most prevalent and lethal cancers in the world, causing a substantial public health burden. The American Cancer Society (ACS) estimates that in 2023, lung cancer claimed the lives of 127,070 men and women in the country, accounting for 238,340 new cases.^1^ Depending on their microscopic morphology, lung tumors are categorized into nonsmall cell lung cancer (NSCLC) and small cell lung cancer (SCLC). New developments in molecular profiling approaches have aided in creating personalized medication based on individual protein or genetic profiles.^2,3^ Consequently, molecularly targeted therapy has emerged as one of the most promising customized treatments for NSCLC patients.^4,5^ This treatment focuses on the proteins, genes, or mechanisms that foster cancer development and prolong its course.^6^ Despite advances in diagnosis and therapy, the outlook for lung cancer patients remains miserable, emphasizing the critical need for innovative therapeutic methods.

There has been a rise in research interest in investigating the use of natural compounds in cancer prevention and treatment. Flavonoids, a broad spectrum of phytochemicals found in plants, have recently received much attention for their appealing biological amenities, particularly their potential role in lung cancer.^7−9^ Flavonoids are a type of secondary metabolites with various chemical structures, including flavones, flavanols, flavanones, and anthocyanins, to name a few. These substances are common in vegetables, fruits, nuts, and beverages such as red wine and tea, all essential components of human diets.^10,11^ According to research into their health benefits, flavonoids have an extensive range of biological activity, ranging from anti-inflammatory and antioxidant characteristics to anticancer capabilities.^12−14^ The biological mechanisms behind flavonoid anticancer activities are complex, involving the control of several signaling pathways. Flavonoids can influence critical cellular processes associated with cancer development and progression by modulating key molecular targets such as nuclear factor-κB, mitogen-activated protein kinases, and phosphoinositide 3-kinase/protein kinase B/mammalian target of rapamycin (PI3K/Akt/mTOR).^15−20^ Likewise, new evidence suggests that flavonoids may have synergistic effects when taken with traditional chemotherapeutic drugs or radiation therapy, potentially increasing the efficacy while decreasing side effects. These findings emphasize the potential of flavonoids as supplementary therapeutics or preventive agents in lung cancer treatment.

After receiving treatment with crizotinib, NSCLC patients are treated with ceritinib (CRT), a strong ALK inhibitor developed and marketed by Novartis under the name ZYKADIA that received FDA approval on April 7, 2014 (BCS Class IV). 150 mg gelatin capsules containing a maximum dose of 750 mg are available for CRT. The solubility of CRT is dependent on the pH of the gastrointestinal tract (GIT) since it is a diprotic base with two basic p*K*_a_ values. CYP3A is metabolized and inhibited *in vitro* in a time-dependent and reversible way. CRT also acts as a substrate for the P-gp efflux transporter, leading to low permeability.^21,22^ The bioavailability of CRT is limited by these characteristics as well as substantial hepatic metabolism, low solubility within the pH range of 5–7.4, and P-gp-mediated efflux. While lipophilic salt was one method used in the literature to increase CRT solubility, permeability problems were not addressed.^23^ The novelty of the current study lies in the integration of a co-former, which enhances solubility by stabilizing CRT’s amorphous form through intermolecular interactions. Furthermore, the selected co-former for co-amorphous material (CAM) preparation can augment CRT’s bioavailability by inhibiting the P-gp efflux transporter. Moreover, we substantiated our experimental methodologies with computational techniques. This multipronged approach is designed to elevate CRT’s solubility and bioavailability, representing a significant stride forward in pharmaceutical formulation.

The selected co-formers have properties that either inhibit or act as substrates for certain enzymes (CYP1A2, CYP3A4, and UGT1A4), which can help increase the concentration of CRT in the bloodstream by preventing its metabolism. To find a suitable co-former that binds more strongly to the CYP enzymes than CRT, computational docking tools were used to virtually screen various co-formers based on their binding affinity to CYP3A4. As assessed by molecular docking scores, the co-formers with the highest binding affinity were further investigated using induced fit docking (IFD) and the prime-MMGBSA tools to investigate their binding mode and energy calculations. Depending on the IFD and prime-MMGBSA results, selected co-formers were subjected to 200 ns MD (molecular dynamics) simulations to examine the drug and co-former molecule’s stability with the enzyme. Naringin (NRG) was chosen as a co-former to prepare CAMs with CRT. In most human diets, NRG is a bioflavonoid, a GRAS (generally regarded as safe) compound. NRG has antioxidant, antiallergic, anti-inflammatory, antitumor, antimutagenic, and antibacterial properties.^24−27^ CRT–NRG CAMs were prepared by using the solvent evaporation method. Differential scanning calorimetry (DSC), Fourier-transform infrared spectroscopy (FTIR), and X-ray powder diffraction (XRPD) were used to characterize the CAMs, and *in vitro* dissolution, SRB assay, AOEB, cell cycle analysis, and *ex vivo* and *in vivo* studies were performed.

## 2 Materials and Methods

### 2.1 Materials

CRT form I (Supporting Information Figure S1A) was kindly provided by MSN Laboratories Pvt., Ltd., based in Hyderabad, India, while NRG (Supporting Information S1B) was purchased from Sigma-Aldrich in Bangalore, India. High-performance liquid chromatography (HPLC)-grade acetonitrile was sourced from Merck Life Science Pvt., Ltd., in Mumbai, India, while HPLC-grade isopropyl alcohol and methanol were acquired from Finar Ltd., in Ahmedabad, India. Analytical grade chemicals such as ammonium acetate, boric acid, hydrochloric acid, sodium hydroxide, phosphoric acid, and KH_2_PO_4_ were obtained from Loba Chemicals in Mumbai, India. Ultrapure water necessary for both sample preparation and chromatographic analysis was obtained from the Millipore water system installed within the department.

### 2.2 Methods

#### 2.2.1 *In Silico* Co-former Screening

##### 2.2.1.1 Molecular Docking and MD Simulation

The Schrödinger molecular modeling suite (2022-4) Maestro version 13.4.134 user interface was used for molecular docking and dynamics studies.^28^

##### 2.2.1.2 Molecular Docking Studies

Molecular docking studies were conducted to elucidate the precise nature of the binding interactions between the NRG ligand and target protein CYP3A4. This allowed us to uncover the specific binding interactions, providing a more comprehensive view of their affinity for each other and the structural configurations involved. For this study, we selected and imported a structure with a resolution of 2.15 Å, identified by its PDB ID: 3UA1.^29,30^ The imported protein structure was thoroughly prepared utilizing the “Protein Preparation Wizard” tool, which involved refinement, modification, and a series of minimization steps, including packing missing loops and chains through the “Prime” tool,^31^ trailed by optimization and minimization. The active site and essential residues were diligently preserved throughout this process, while nonprotein water molecules beyond 5 Å were carefully eliminated. Subsequently, another tool called “Receptor Grid Generation” was applied to generate the receptor grid necessary to perform a molecular docking study. The “LigPrep” tool^32^ in Maestro was also utilized to prepare the ligand (NRG) for the investigation, with “Epik” acting as the ionization tool and OPLS4^33^ acting as the force field. Finally, the docking studies were executed in Maestro, employing the “Glide”^34^ module, which operated in extra-precision (XP) mode.

In XP docking, the receptor’s flexibility is constrained, failing to accurately mimic the dynamic nature of biological processes where proteins and ligands interact within a solvated environment. To address this limitation, we performed an MD simulation. Subsequently, the protein–ligand complexes obtained from the docking study were used as the starting point for these comprehensive MD simulation studies, allowing for a more realistic exploration of their interactions in a solvated context.

##### 2.2.1.3 MD Simulation

MD simulations are essential for gaining insights into the functionality and dynamics of both protein and protein–ligand systems. To explore deeper into the dynamic behavior of the protein when interacting with a ligand, we performed MD simulations using the “Desmond” tool, integrated with Maestro.^35,36^ This multistep MD simulation process comprises system building, minimization, and finally dynamics simulation. For the MD simulation, we utilized the OPLS4 force field. During the system building phase, we employed the simple point charge solvent model, placing the system within an orthorhombic box with box size measured using the buffer method. To maintain charge neutrality, sodium and chloride ions were added. Subsequently, we conducted energy minimization and equilibration procedures for the prepared system, maintaining a temperature of 300 K and a pressure of 1.01325 bar within the *NPT* ensemble. The MD simulation extended 200 ns, with trajectory recording intervals set at 200 picoseconds (ps). The resulting simulation data was comprehensively analyzed using Desmond’s “Simulation Interaction Diagram” tool, offering a detailed understanding of the system’s dynamic behavior.^37^

#### 2.2.2 Preparation of CRT–NRG Co-amorphous Systems

The solvent evaporation preparation technique was employed for the preparation of CAMs. 50 mL of methanol was added to a uniformly mixed physical blend of drugs and co-formers, resulting in a clear solution. The solvent was then removed using a rotary evaporator under reduced pressure at 40 °C, yielding a transparent film of the product. This film was scraped from the round-bottom flask and transferred to a desiccator filled with CaCO_3_, where it was placed under vacuum for 24 h to eliminate any remaining solvent. CAMs of CRT and NRG mixtures prepared in the following molar ratios: 1:1 (CRT 245.07 and NRG 254.91 mg), 1:2 (CRT 162.30 and NRG 337.64 mg), and 2:1 (CRT 328.85 and NRG 171.03 mg) of a total 500 mg.^38^

#### 2.2.3 Characterization of CAMs

##### 2.2.3.1 Differential Scanning Calorimetry

The Shimadzu-DT-60 DSC equipment was utilized to obtain thermograms of various samples including CRT, NRG, a physical mixture of CRT–NRG (CNPM), and CAMs. For the thermal analysis, each sample powder of approximately 5 mg was securely sealed in a flat-bottomed aluminum pan by using an aluminum lid. These pans containing the samples were then positioned in a sample holder and subjected to heating at a rate of 10 °C per minute, starting from 20 to 220 °C. A constant flow of nitrogen gas (at a rate of 10 cm^3^/min) was maintained around the samples.

The instrument temperature was calibrated with an indium standard, maintaining the same heating rate and pan type as those described earlier. Additionally, the heat flow and heat capacity signals were calibrated using powdered alumina (5 mg, 100 mesh) as a reference. To verify consistency, the sample was tested in triplicate (*n* = 3) for reproducibility. Furthermore, the glass-transition temperature (*T*_g_) was evaluated from the relevant DSC thermogram by considering the midpoint value.

##### 2.2.3.2 Powder X-ray Diffraction

Diffractograms of the CRT, NRG, CNPM, and CAMs samples were captured by using a Rigaku MiniFlex 600 X-ray diffractometer. The instrument utilized 600 W, with an X-ray tube voltage of 40 kV and a constant tube current of 15 mA. The detector employed in the apparatus was a conventional scintillation counter equipped with a graphite monochromator. Intensity measurements were conducted in the 5–40° (2θ) range using a fixed-time step scanning method.

##### 2.2.3.3 FTIR

FTIR spectra were obtained using a Shimadzu spectrophotometer model IRAffinity-1S. The spectral range covered was 4000–400 cm^–1^, and each spectrum was recorded by averaging 25 scans with a resolution of 2 cm^–1^. To prepare the samples for analysis, they were mixed with KBr and ground to a fine powder using a mortar and pestle. The resulting mixture was then compressed under a pressure of approximately 1000 psig to form a disc shape before being subjected to FTIR measurements.

#### 2.2.4 Miscibility (Drug–Coformer)

Drug–coformer miscibility was calculated using the Gordon–Taylor equation and solubility parameter approaches. Based on experimental DSC data, theoretical predictions of the glass transition temperatures for different CAMs were made using the Gordon–Taylor equation. The solubility parameters (δ) for CRT and NRG were calculated by using the van Krevelen group contribution method. This approach considers cohesive energy as an indicator of the attractive forces between molecules in a substance.^38^

#### 2.2.5 Physical Stability

CRT–NRG CAMs in varied molar ratios (1:1, 1:2, and 2:1) were placed in a desiccator that contained CaCO_3_ crystals. CAMs in the desiccator were monitored for the onset of crystallization at 25 °C for up to 6 months. After the storage time, the samples were analyzed using XRPD.

#### 2.2.6 Solubility

##### 2.2.6.1 Thermodynamic Solubility

The shake flask method was used to determine the solubility of CRT, CNPM, and CRT–NRG CAMs utilizing USP pH 6.8 phosphate buffer. Excess amounts of CRT and CAMs were added to solubility vials (2 mL) containing the buffer. These vials were then subjected to agitation on an orbital shaker for 24 h at 37 °C and 120 rpm. After this incubation period, the pH of the samples was checked and adjusted if necessary just before centrifugation. Centrifugation was carried out at 37 °C, 10,000 rpm, for 10 min, trailed by the collection of the supernatant and its dilution using the mobile phase. The samples were then analyzed by using the HPLC method described below. This experimental procedure was conducted in triplicate (*n* = 3).

##### 2.2.6.2 Amorphous Solubility

The amorphous solubility of CRT was measured using the solvent quench method, which employed methanol and HPMC as the solvent and crystallization inhibitor, respectively. Methanol was used as the primary solvent for preparing a highly concentrated stock solution (10 mg/mL) owing to the drug’s solubility properties. The detailed amorphous solubility methodology can be found in Shete et al. (2021).^39^

##### 2.2.6.3 HPLC Method

The Shimadzu Prominence UHPLC with a UV detector was used for the analysis. A GraceSmart Brava BDS-C_18_ column (250 mm × 4.6 mm × 5 μm) was used for chromatographic separation. The mobile phase was given in a gradient manner with 42:50:8% v/v methanol:25 mM phosphate buffer pH 3.0:ACN for 5 min and 50:50% v/v acetonitrile:25 mM phosphate buffer pH 3.0 for 17 min at a flow rate of 1 mL/min. At 4 and 25 °C, the autosampler and column temperatures were maintained consistently with detection wavelengths of 275 (CRT) and 288 (NRG).

#### 2.2.7 Biorelevant Dissolution

Powder dissolution tests were conducted on CRT and CAMs samples by placing 150 mg of each substance (equivalent to 150 mg of CRT) into 60 mL of fasted-state simulated intestinal fluid (FaSSIF) and fed-state simulated intestinal fluid (FeSSIF) provided by a Biorelevant company. The suspension was stirred continuously at 37 °C and 120 rpm using a magnetic stirrer. Samples were collected at regular intervals of 15, 30, 60, 90, and 120 min, followed by centrifugation at 10,000 rpm and 4 °C for 10 min. The supernatant was then collected for analysis using the specified HPLC method.

#### 2.2.8 *In Vitro* Cell Line Studies

##### 2.2.8.1 SRB Assay (Sulforhodamine B)

The cytotoxicity of CRT, NRG, and CAMs was evaluated by using the sulforhodamine B (SRB) colorimetric assay. A cell suspension was made based on cell count, and 5000 cells were seeded per well in a 96-well plate. After 24 h, the cells were treated with seven different concentrations of CRT and NRG: 50, 25, 12.5, 6.25, 3.125, 1.56, and 0.78 μM. The SRB assay was performed after a 48 h incubation at room temperature, and the absorbance at 540 nm was determined using a plate reader.^40^

##### 2.2.8.2 AO/EB Staining (Acridine Orange/Ethidium Bromide)

A549 cells were seeded in six-well plates at a density of 3 × 10^6^ cells. After allowing the cells to attach for 24 h and form a monolayer, they were treated with CRT, NRG, and CAMs at the previously determined IC_50_ dose. The cells were then incubated at room temperature for 24 h. After incubation, the cells were washed three times with freshly prepared phosphate-buffered saline. Next, 1 mL of ethanol was added to each well for fixation, and the cells were incubated for 10 min. A solution of AO/EB (1 mL per well) staining was added to each well and incubated for another 10 min. The cells were examined morphologically using an inverted microscope fitted with fluorescence filters.^41^

##### 2.2.8.3 Cell Cycle Analysis

Exponentially growing cells were detached from T-25 cell culture flasks using trypsin and then transferred to sterile Petri plates. The cells were allowed to attach and grow for approximately 24 h. After the attachment period, different treatments were applied to the cells, and the plates were further incubated for 48 h. The media was removed after 48 h of incubation, and the plates were rinsed two times with phosphate buffer. After that, the cells were trypsinized and centrifuged at 4 °C. After centrifugation, the pellet was fixed in 70% ice-cold ethanol for 30 min at 4 °C. The resulting pellet was resuspended in Dulbecco’s phosphate-buffered saline (PBS) to wash the cells. After another round of centrifugation, the supernatant was removed and the pellet was redispersed in a staining solution consisting RNase, propidium iodide, and NP-40. For 20 min, the cell suspension was incubated in the dark at room temperature. The cells were then analyzed with a BD Accuri C6 flow cytometer (BD Biosciences, San Jose, CA, USA).^42^

#### 2.2.9 *Ex Vivo* Studies

The *ex vivo* assessment complied with the guidelines provided by the Committee for the Purpose of Control and Supervision of Experiments on Animals (CPCSEA). The Manipal Academy of Higher Education Institutional Animal Ethics Committee (MAHE-IAEC, IAEC/KMC/24/2020) approved the study. Wistar rats weighing 220 ± 10 g were subjected to a 12 h fasting period before the experiment. The ileum was obtained by euthanizing the animals through cervical dislocation and making a midline incision in the abdomen to extract the small intestine. After the ileum was washed thoroughly with saline solution, it was retrieved by making a cut 5 cm above the ileocecal junction. The ileal segment was everted, washed with saline solution, and used to measure the permeability from the mucosal to the serosal side using a capillary tube. A sealed compartment was fashioned by tying off the proximal end to a receptor silicone tube and securing the distal end with a thread. The detailed *ex vivo* methodology can be found at Yarlagadda et al. (2023).^38^

### 2.3 Pharmacokinetic Studies

Pharmacokinetic studies were conducted by following the CPCSEA guidelines. MAHE-IAEC (IAEC/KMC/24/2020) approved the study. The study utilized a parallel design, with each group comprising 3 male Wistar rats weighing approximately 200 g, with a variation of ±20 g. Prior to treatment, the rats underwent a 12 h fasting period but were allowed access to water. Treatment A involved administering a dose of 15 mg/kg of CRT, suspended in a 0.5% (w/v) carboxyl methyl cellulose (CMC) aqueous solution. Treatment B consisted of a 15 mg/kg equivalent CRT CAMs suspension prepared in a 0.5% (w/v) CMC vehicle. The animals received 1 mL of the suspension orally via oral gavage. The interested readers are directed to the pharmacokinetic study reported by Yarlagadda et al. (2023) for detailed methodology.^38^

## 3 Results and Discussion

The objective of the present study was to identify a co-former molecule for the preparation of co-amorphous systems with CRT. This molecule should possess the ability to bind to the enzyme, acting either as a substrate or inhibitor, with a binding affinity surpassing that of CRT. This would result in increased free CRT concentration in the plasma, enhancing its availability for absorption. This study considered 32 GRAS compounds as co-formers. Each co-former was individually docked onto the CYP3A4 protein, and the selection criteria involved higher binding affinity and greater intermolecular interactions. Notably, NRG stood out as the sole molecule displaying the highest binding affinity, reflected in a docking score of −10.970 kcal/mol and a binding energy of −39.69 kcal/mol with the CYP3A4 (Supporting Information Tables S1 and S2).

A variety of factors, including protein–ligand complex stability, binding mode prediction, the nature of interactions with the CYP3A4 binding site, and NRG, were investigated using MD simulations Supporting Information (Figure S2 and Table S2). Throughout the simulation, a frame was taken every 20 ps and saved onto a trajectory. The simulation exercise yielded approximately 1000 frames in total. Information regarding structural deviation and protein stability is provided by the parameter root-mean-square deviation, or RMSD.

**Figure 1:**
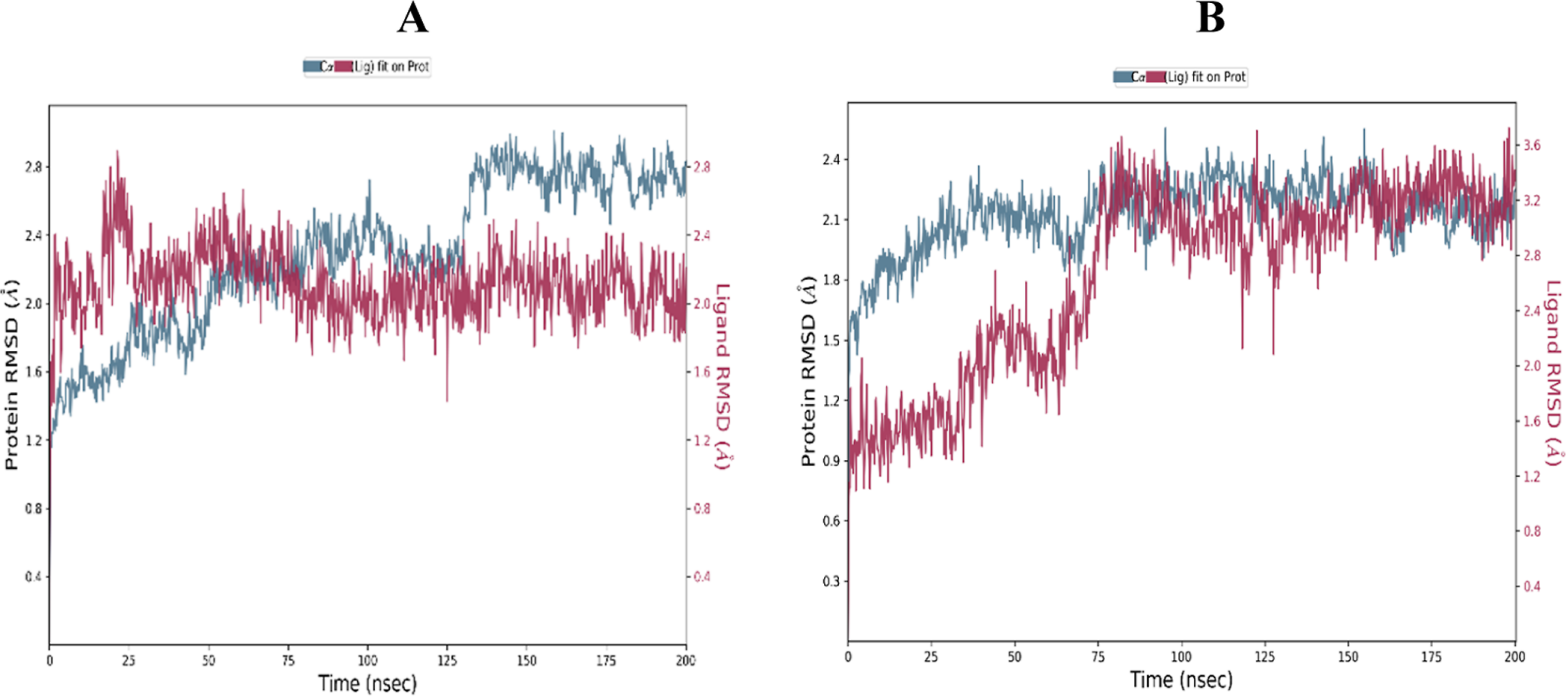
RMSD is in the presence of CRT (A) and NRG (B) with CYP3A4.

The RMSD of CYP3A4 in the presence of CRT (A) and NRG (B) is displayed in Figure 1. It became apparent that the CRT RMSD was rather steady between the 80 and 200 ns simulation periods. In contrast, the NRG experienced a small drift throughout the first stages of the simulation before staying constant the entire time.

**Figure 2:**
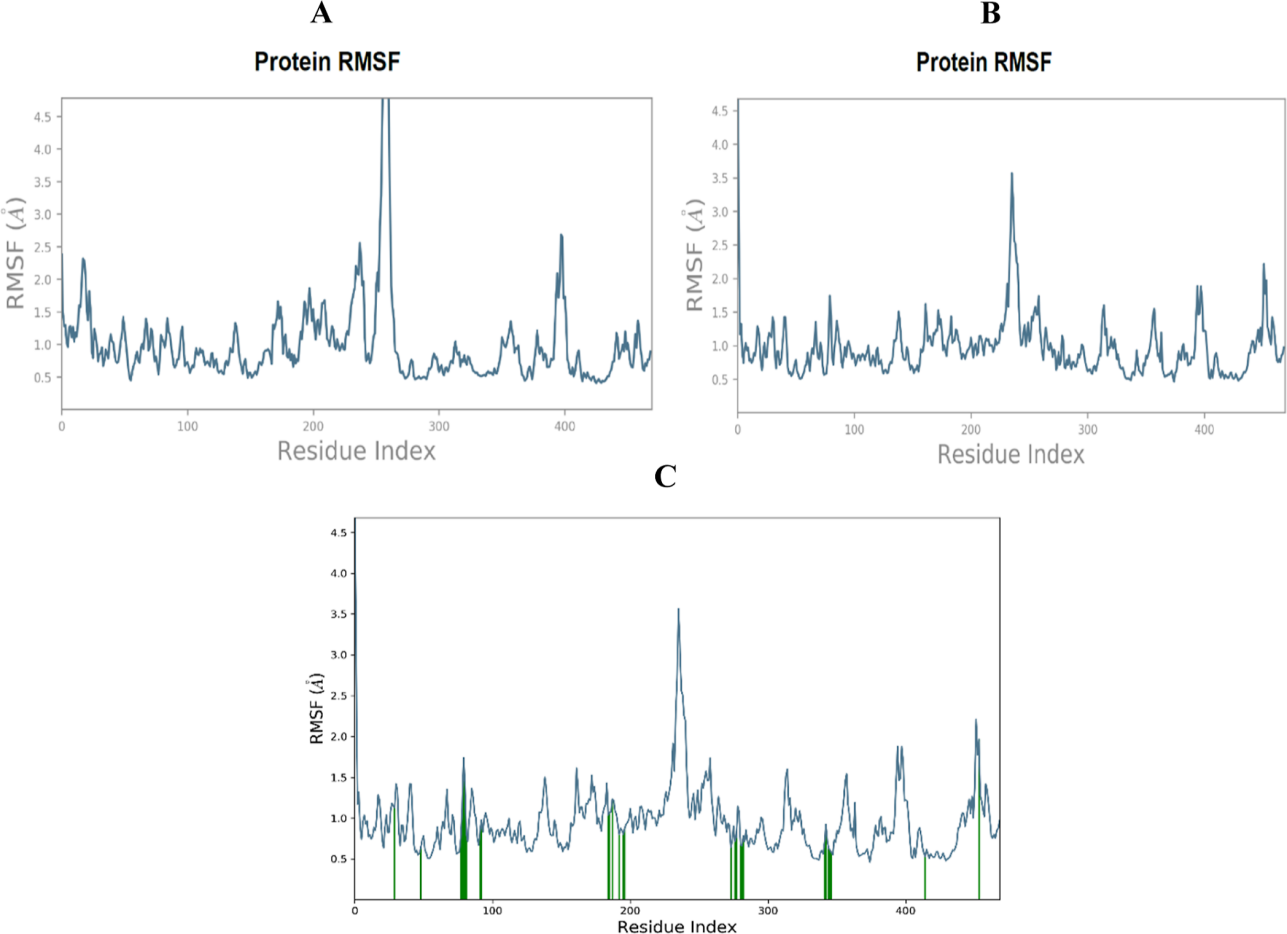
Protein RMSF in the presence of CRT (A), NRG (B), and NRG contact regions in protein RMSF.

To determine the residue-wise variations of the protein across the simulation period, root-mean-square fluctuation (RMSF) was defined. Residue variations are the subject of the RMSF study, highlighting their significance since CYP3A4 would be impacted by any abrupt alterations in the functionally relevant residues’ flexibility. Figure 2 displays the general variations in the CYP3A4 residues in the presence of CRT and NRG and the ligand contact sites in the RMSF plot. Regarding the CRT, the amino acid residues of CRT–CYP3A4 from 0 to 250 have shown very few fluctuations in the RMSF plot; however, the area of protein involving amino acid 251–285 and mostly residues GLU_262, ASP_263, THR_264, and GLN_265 showed large *B*-factors as well as fluctuations in RMSF as can be observed in Figure 2A. On the other hand, minimal fluctuation was seen in the first region where NRG was present, and some subsequent oscillations were observed in the region between 230 and 250.

In Figure 2C, the protein’s RMSF plot highlighted regions of ligand contact, specifically between amino acid residues 50 to 100, 100 to 200, and 200 to 300, along with 350 to 450 (indicated in green). Further analysis aimed to understand the consistency of the protein–ligand interactions throughout the simulation process.

**Figure 3:**
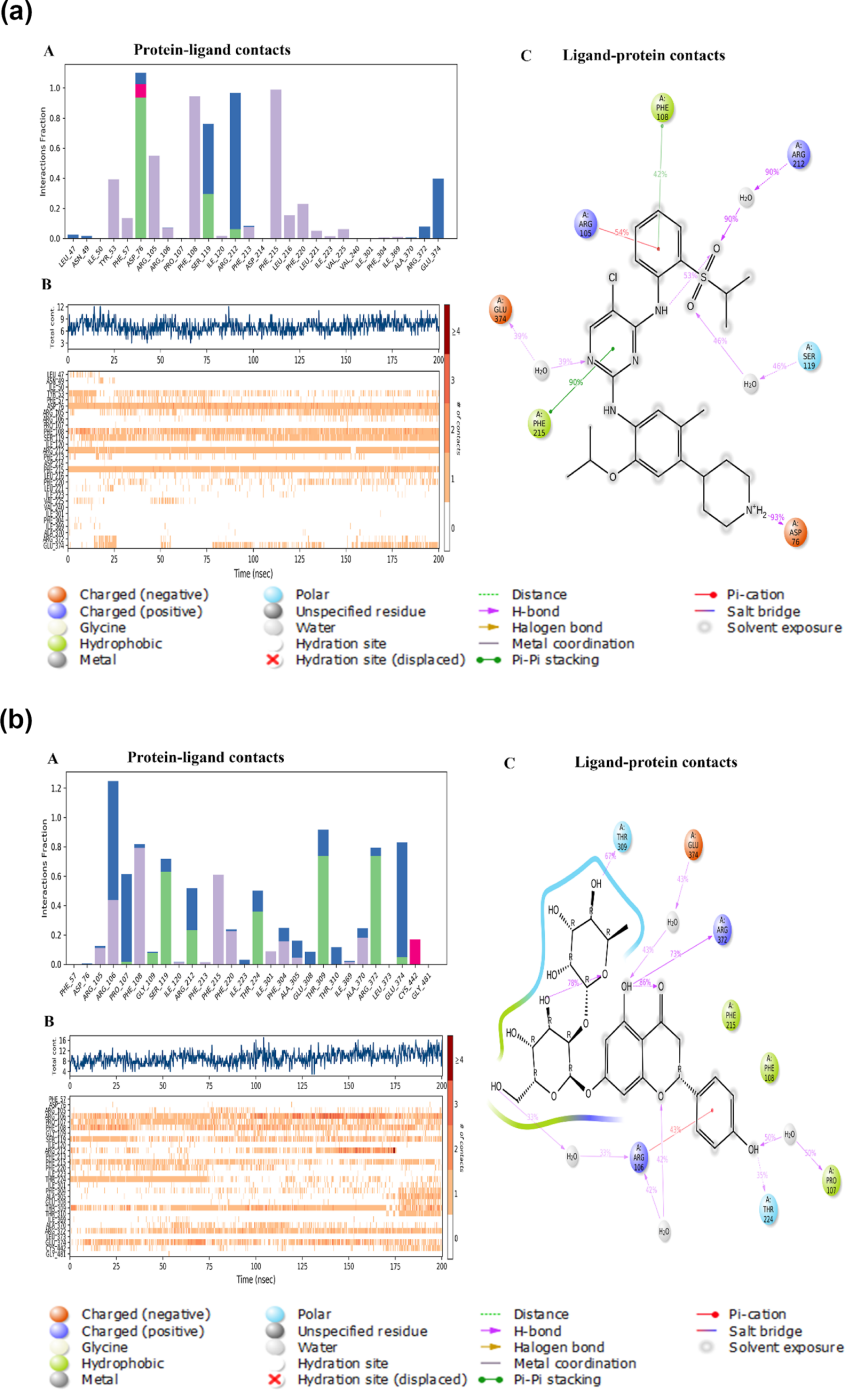
(a) Figures illustrating protein–ligand interactions between CRT and CYP3A4 derived from MD simulation. (A) Graph showing the frequency of various protein–ligand interactions between CRT and various CYP3A4 amino acids. (B) Time line depicting the interactions between CYP3A4 and CRT (H-bond contacts and interactions shown vs time). (C) A two-dimensional ligand interaction map shows interactions that happen for over 30 percent of the 200 ns simulation time; the purple color arrow indicates a hydrogen bond with amino acids (for an explanation of the color references in this figure legend, the reader is referred to the article’s online version). (b) Figures illustrating protein–ligand interactions between NRG and CYP3A4 derived from MD simulation. (A) Graph showing the frequency of various protein–ligand interactions between NRG and various CYP3A4 amino acids. (B) Time line depicting the interactions between CYP3A4 and NRG (H-bond contacts and interactions shown vs time). (C) A two-dimensional ligand interaction map shows interactions that happen for over 50 percent of the 200 ns simulation time; the purple color arrow indicates a hydrogen bond with amino acids (for an explanation of the color references in this figure legend, the reader is referred to the article’s online version).

A comprehensive report presented in Figure 3 detailed potential interactions, revealing multiple water bridge formations between NRG and amino acid residues such as ARG 106, PRO 107, SER 119, ARG 212, THR 224, PHE 304, ALA 305, GLU 308, THR 309, THR 310, ILE 369, ALA 370, and LEU 373, in contrast to CRT, which showed interactions with SER 119, ARG 212, ARG 372, and GLU 374 [Figure 3a(A)]. The temporal dynamics of interactions between CRT and CYP3A4, as well as NRG and CYP3A4, were depicted on a timeline, with hydrogen-bond interactions plotted against time, where darker colors denoted stronger contacts [Figure 3a(B)]. Specifically, NRG consistently engaged in seven hydrogen-bond interactions with amino acids PHE 108, GLY 109, ILE 120, THR 224, THR 309, ARG 372, and GLU 374, persisting for over 50% of the simulation duration [Figure 3b(C)]. Overall, the results of the MD simulation affirmed the stability and stronger binding interactions of the NRG-CYP3A4 protein–ligand complex compared with CRT–CYP3A4, involving distinct amino acids.

**Figure 4:**
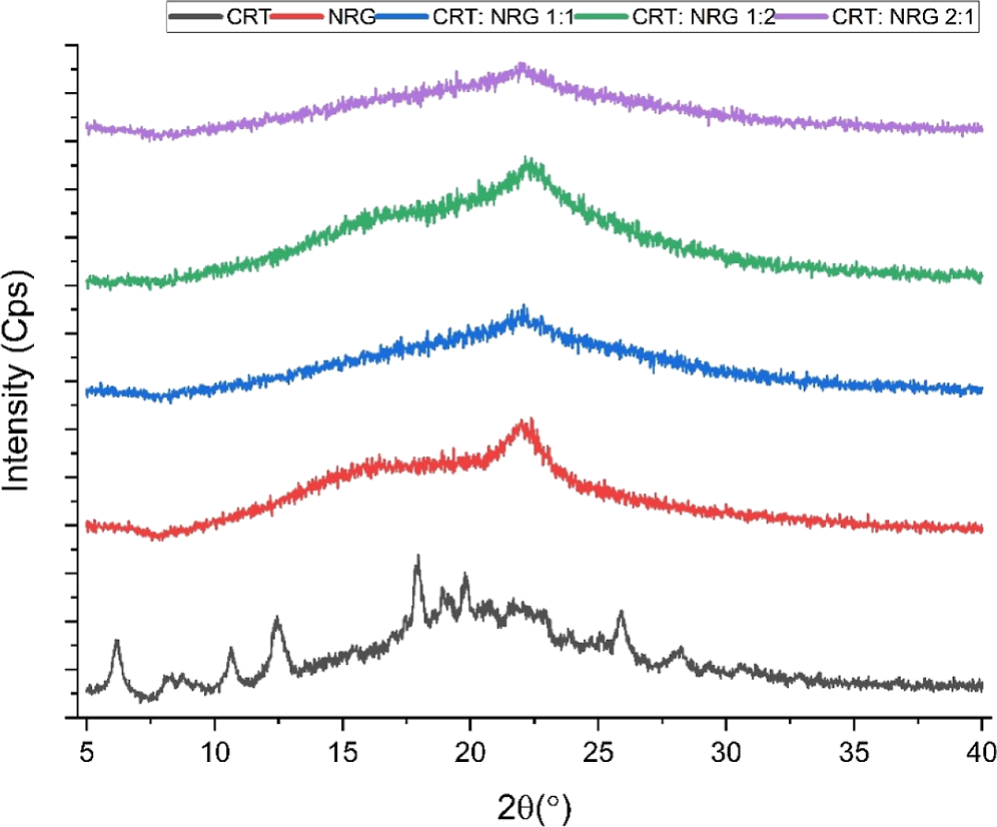
XRPD of CRT, NRG, and CAMs.

### 3.1 Solid-State Characterization

XRPD analysis of the CAMs samples was carried out to corroborate the transition from the crystalline to amorphous form. CRT exhibited intense Bragg’s diffraction at 6.14, 10.68, 12.42, 17.92, and 25.86°, confirming the crystalline nature. Regardless of the molar ratio, a diffuse halo pattern was observed in all CAMs, indicating the lack of the crystalline phase (Figure 4).

**Figure 5:**
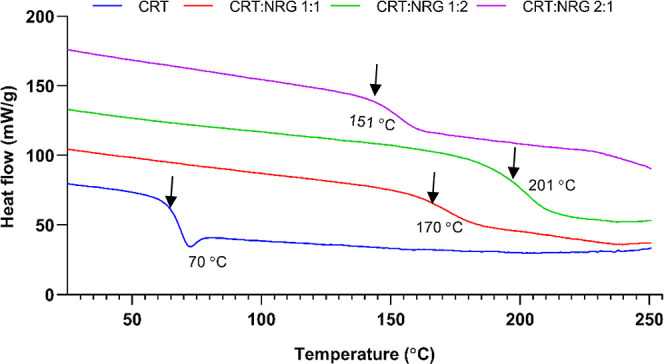
DSC thermograms of amorphous CRT and its corresponding CAMs.

This study investigated the thermal behavior of co-amorphous systems using DSC and compared them to physical mixtures and individual compounds of CRT and NRG. It was found that CRT and NRG exhibited endothermic and glass transition events at temperatures of 170^38^ and 167 °C,^43^ which implies the crystalline and amorphous nature. All CAMs of CRT and NRG exhibited a single glass transition temperature (*T*_g_), indicating the formation of a homogeneous single phase (Figure 5). Typically, in a homogeneous amorphous system with no specific intermolecular interactions, the *T*_g_ value falls between the *T*_g_ values of the individual compounds. This behavior can be calculated using the Gordon–Taylor equation.^44^ However, deviations from this equation can occur if there are interactions between the compounds in the co-amorphous system. In this study, a positive shift in *T*_g_ values was reflected for the CRT-NRG coamorphous systems, with the highest shift seen in the CRT:NRG 1:2 system at 73 °C, followed by CRT:NRG 1:1 at 59 °C, and CRT:NRG 2:1 at 53 °C, than the calculated *T*_g_ values presented in the Supporting Information (Figure S3). This implies the likelihood of intermolecular interactions between CRT and NRG in the coamorphous systems.

**Figure 6:**
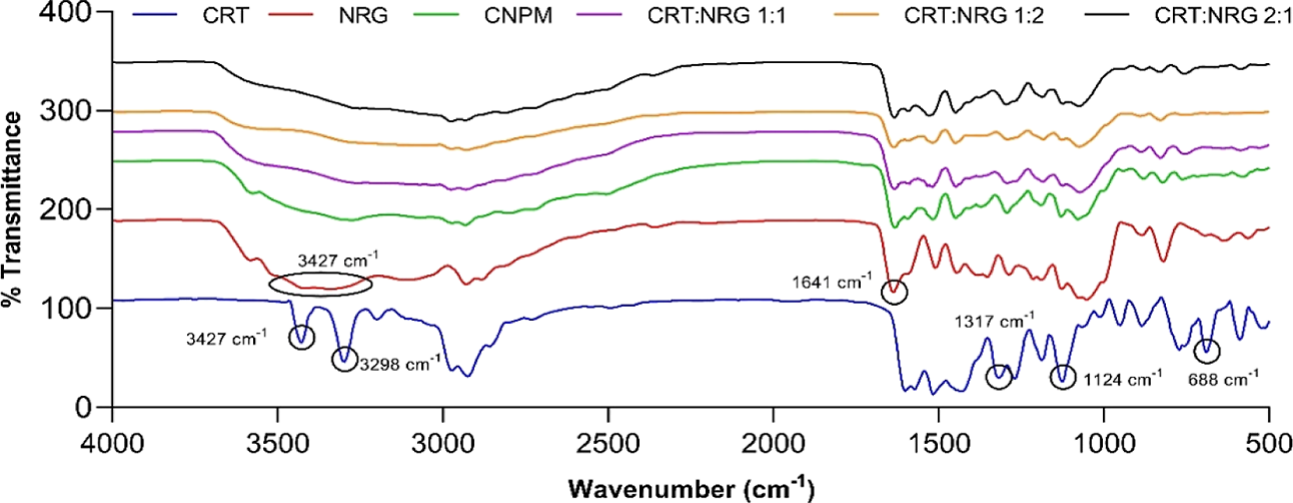
FTIR spectra of CRT, NRG, CNPM, and their corresponding CAMs.

Molecular-level interactions like intermolecular and intramolecular interactions are critical for co-amorphous system stability. FTIR studies were conducted to examine the interactions between the CRT and NRG. The IR spectrum of CRT consists of sharp peaks at 3427 and 3298 cm^–1^, which signifies the aromatic and aliphatic secondary amine (N–H) stretching. Furthermore, peaks at 1124 and 1317 cm^–1^ depict the typical symmetric and asymmetric sulfone stretch vibrations. In addition to these vibrations, C–Cl’s strong stretching is apparent in the spectrum of CRT at 688 cm^–1^. The NRG spectrum showed a characteristic sharp peak at 1641 cm^–1^, attributed to the C=O group. At 3427 cm^–1^, a broad peak was distinguished for the presence of the OH group. Additionally, peaks at 2928 and 2885 cm^–1^ were observed for sp^3^-hybridized alkyl stretching (Figure 6).

**Figure 7:**
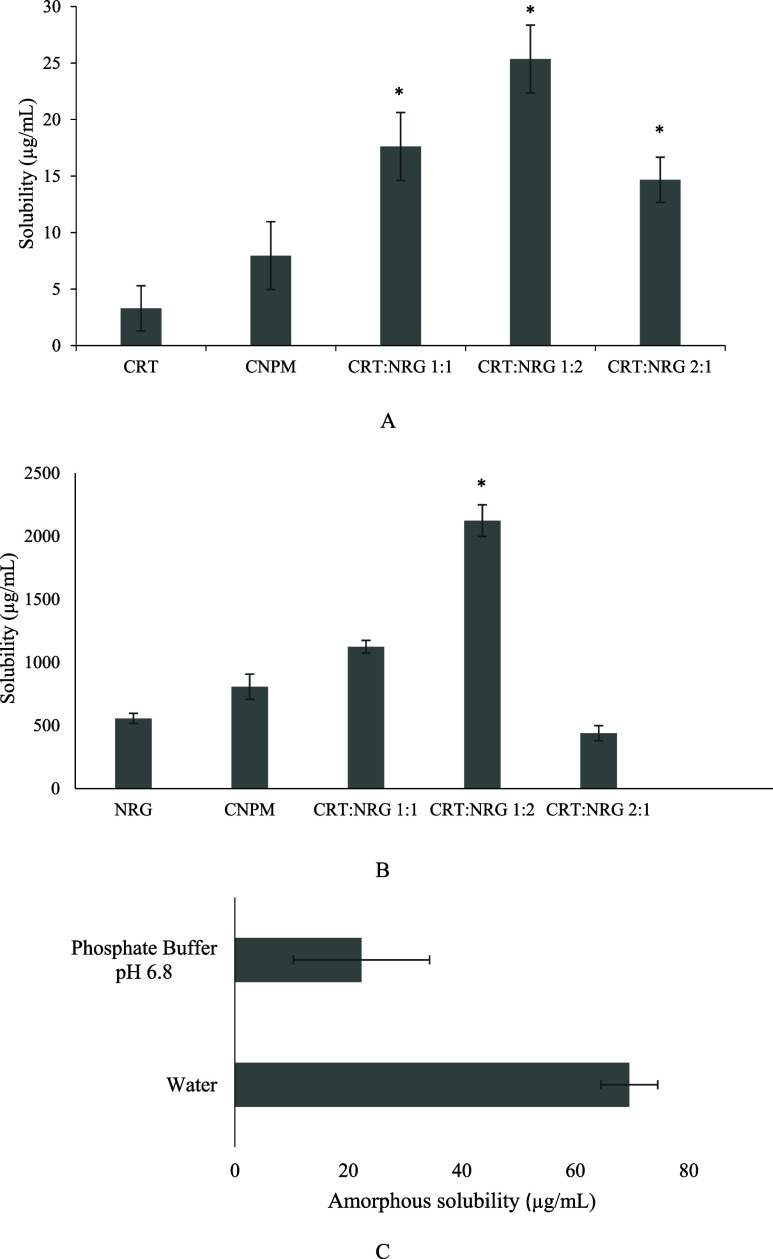
(A) Thermodynamic solubility of CRT, CNPM, and CAMs. (B) Thermodynamic solubility of NRG, CNPM, and CAMs. (C) Amorphous solubility of CRT in water and phosphate buffer. * implies a substantial difference when compared to the CNPM (control). *p* < 0.05 significantly different.

The CRT and NRG physical mixture (CNPM) spectrum was compared to those of prepared CAMs to acquire insights into intermolecular interactions (if any). The transition from crystalline to amorphous chemical environments is reflected in the diminution of peaks in the spectra of all coamorphous CRT-NRG systems compared to their respective physical mixtures. However, the N–H stretching bands at 3427 and 3298 cm^–1^ vanished completely in all spectra of CAMs compared to the physical mixture. A substantial shift was observed in the case of sulfone stretch vibrations at 1124 cm^–1^ to a higher frequency of 1126 cm^–1^ in CAMs. Furthermore, carbonyl stretching in NRG at 1641 cm^–1^ was attenuated in all of the CAMs without any considerable shift. Interestingly, the C–Cl vibration at 688 cm^–1^ vanished from the CAMs. The absence of characteristic N–H and C–Cl bands in CAMs indicates the presence of specific hydrogen bonds between the N–H group of CRT and the OH group of NRG. As revealed by FTIR spectroscopy, this observation is further supported by Gordon–Taylor calculations, which elucidate intermolecular interactions between CRT and NRG upon amorphization. Computational studies employing MD simulations were conducted to visualize these intermolecular interactions. The simulations illustrated the formation of hydrogen bonds between CRT and NRG, as provided in the Supporting Information (Figure S4), thereby confirming the presence of significant intermolecular interactions, particularly through hydrogen bonding, between CRT and NRG.

### 3.2 Drug–Coformer Miscibility

Drug–coformer miscibility is a critical prerequisite for coherent formulation design, especially when evaluating CAMs. A thoroughly dispersed and molecularly disseminated drug within a coformer is quite enviable. This will inhibit supersaturation-driven recrystallization or phase separation in the liquid and solid states. As a result, the phase solubility of the drug and coformer pair is imperative which can be determined by computational, theoretical, and experimental approaches.^39^ Van Krevelen’s contribution values were considered to calculate drug–coformer miscibility by the Hildebrand and Hansen technique. The difference in Hildebrand solubility parameters of CRT and NRG is <7 MPa^1/2^, indicating that the system is single phase. The dispersibility (δd), hydrogen bonding (δh), and polarity (δp) contributions are factored in when calculating the Hansen solubility parameter (Table 1). The CRT–NRG pair values are relatively close for δd, whereas δp and δh show a substantial difference. Nevertheless, the CRT–NRG co-amorphous system is expected to be single phase when considering each structure’s overall group contributions. Binding energies for CRT and NRG were also computed to ascertain miscibility. The observation of negative binding energy suggested that the CRT–NRG coamorphous system had blended entirely to produce a single-phase system.

**Table 1:** Drug Coformer Miscibility Calculation Using the Hildebrand and Hansen Solubility Parameter Method

s.no.	substance	molecular weight (g/mol)	density (g/cm^3^)	molecular volume (cm^3^/mol)	melting point (K)	Δ*H* fusion (kJ/mol)	solubility parameter (MPa^1/2^)			
							Hildebrand	Hansen		
								δd	δp	δh
1	ceritinib^38^	558.14	1.3 ± 0.0.1	423.18	442.14	15.13	32.78	14.48	4.23	8.13
2	naringin^45^	580.54	1.5 ± 0.0.1	372.14	440.15	2.09	30.63	18.21	12.02	21.69

### 3.3 Thermodynamic Solubility

The thermodynamic solubility was estimated in a phosphate buffer of 50 mM (pH 6.8). Figure 7 depicts the solubility results. For 24 h, the thermodynamic solubility of CRT was observed as 3.3 μg/mL. CRT solubility has improved significantly in the physical mixture and in all CAMs. The CRT:NRG 1:2 exhibited an eightfold improvement, followed by CRT:NRG 1:1 (5.3-fold) and CRT:NRG 2:1 (4.4-fold), particularly in the thermodynamic solubility of CRT. An analogous trend was shown in the case of NRG equilibrium solubility, where NRG solubility was improved in all of the CAMs except for 2:1 CRT:NRG 2:1. CRT:NRG 1:2 CAMs showed the most significant improvement of 3.8-fold in solubility. The improvement in the solubility of CAMs is attributed to the presence of hydrogen bonds between CRT and NRG.

**Figure 8:**
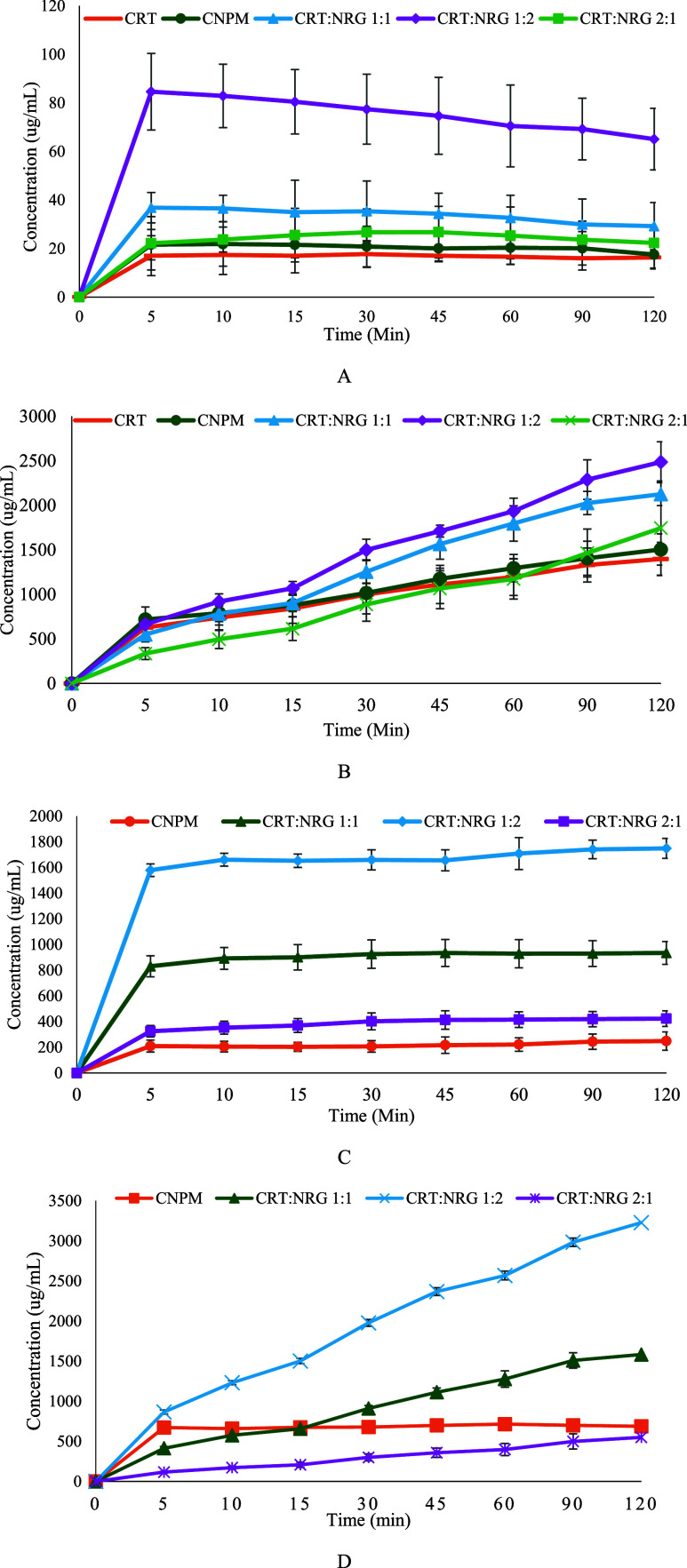
(A) FaSSIF dissolution profiles of CRT, CNPM, and CAMs. (B) FeSSIF dissolution profiles of CRT, CNPM, and CAMs. (C) FaSSIF dissolution profiles of NRG, CNPM, and CAMs. (D) FeSSIF dissolution profiles of NRG, CNPM, and CAMs (*n* = 3, *p* < 0.05, show substantial differences in dissolution between the physical mixture and CAMs CRT:NRG 1:2) for all FaSSIF and FeSSIF profiles.

The amorphous solubility of CRT was also evaluated using distilled water and pH 6.8 phosphate buffer (Figure 7C). Interestingly, the amorphous solubility of CRT is approximately threefold higher in distilled water than in the phosphate buffer. The discrepancy of CRT solubility in phosphate buffer may be ascribed to the salting out of CRT, unlike that in distilled water. This effect occurs when phosphate salt causes an increase in the ionic strength of the solution. Furthermore, this reduces the contact area with aqueous solvent by forming CRT aggregates of hydrophobic surfaces. As a result, the solubility of CRT decreases with rising concentration of salt. The phenomenon is commonly employed for protein purification but has also been observed with small molecular weight compounds such as ritonavir and some polymers. Additionally, the increased solubility of CRT with NRG in CNPM may be attributable to the *in situ* amorphization of CRT in the presence of NRG, which is facilitated by water molecules. Due to their many hydrogen bonding sites, CRT and NRG are pliable for *in situ* amorphization. Ojarinta et al. (2016) observed a similar trend with the physical mixture of indomethacin (IND) and arginine (ARG). *In situ* amorphization was observed due to the interaction between IND aromatic and ARG guanidium groups.^46^ An alternative rationale for this behavior is the formation of phosphate aggregates with NRG, which is plausible when the anionic form of acidic NRG predominates in the solution and can thus form complexes with organic cations when the pH of the solution increases. Few studies have been published in which weakly basic molecules’ solubility altered drastically as a result of the development of phosphate aggregates when exposed to phosphate buffers. Chegireddy et al. (2020) studied the influence of quercetin (QCT) on saquinavir’s solution-phase behavior. They observed the reduction in saquinavir concentration due to the formation of phosphate aggregates with QCT.^47^ KS et al. (2022) reported a significant decrease in raloxifene solubility in the presence of QCT when treated with phosphate buffer pH 6.8. The exposed QCT sample was analyzed using DSC, which revealed a change in QCT *T*_g_, indicating the development of phosphate aggregates.^48^

### 3.4 Biorelevant Dissolution

The dissolution rate of CRT was remarkably higher for CAMs than the CRT. The biorelevant dissolution results of pure CRT, CNPM, and CAMs are depicted in Figure 8. The enhancement in the dissolution of CRT for CAMs is approximately fourfold higher than in the pure crystalline form. The dissolution results correspond to the solubility profiles where CRT:NRG 1:2 showed maximum release and, subsequently, CRT:NRG 1:1 and CRT:NRG 2:1 CAMs. Furthermore, the dissolution of NRG also became apparent in improvement, which is substantially improved than CNPM and pure NRG; the most significant is sevenfold (CRT:NRG 1:2) CAM.

**Figure 9:**
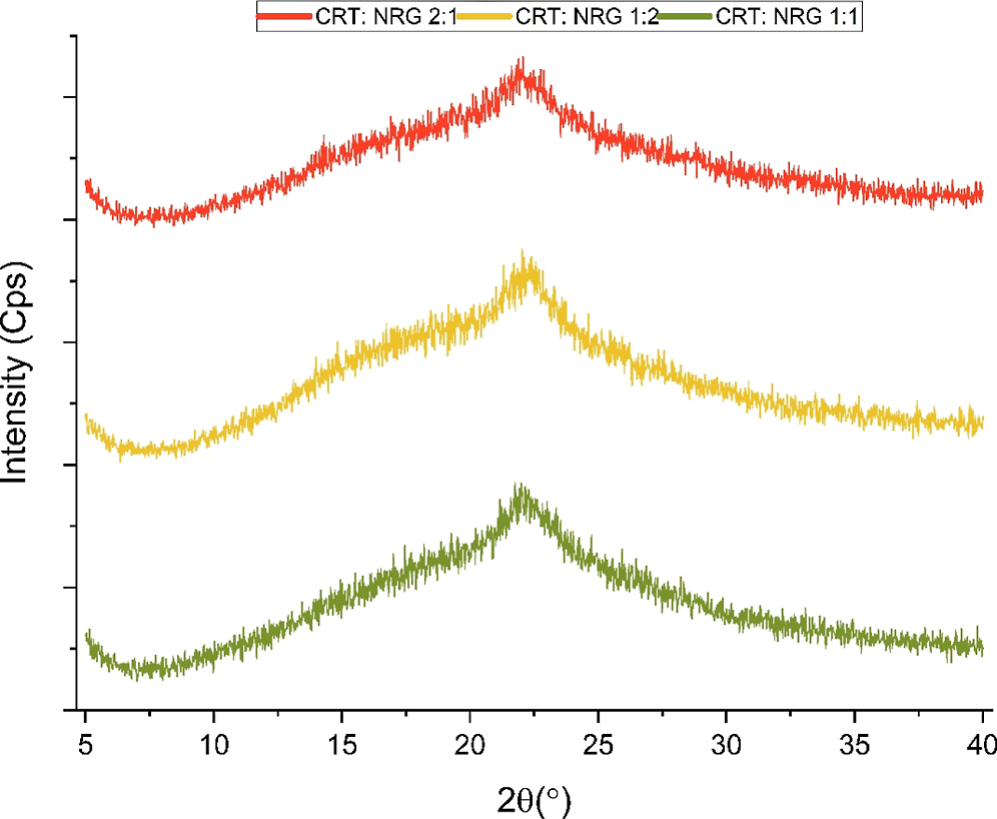
XRPD of physical stability at 40 °C for 180 days exposed to CAMs.

The improvement in CRT dissolution was significant only in the case of CRT:NRG 1:2 among all CAMs. The rationale for improved dissolution of CRT may be ascribed to the multifacet mechanisms, including the presence of hydrogen bonds, facile salt formation, amorphization, and micellar solubilization. The facile salt formation involves the weakly basic CRT and weakly acidic NRG through ionic interactions,^49^ specifically the interaction between phenolic hydroxyls in NRG flavone and glycosidic parts with CRT’s piperidine and pyrimidine nitrogen or sulfone parts. Ojarinta et al. (2016) prepared IND co-amorphous systems using amino acids to inhibit crystallization. The enhancement in the IND dissolution rate was reported due to co-amorphous salt formation between IND and ARG.^46^ Furthermore, Kasten et al. (2018) also described the co-amorphous salt formation between naproxen and ARG CAMs.^50^ Additionally, hydrate formation may aid in improved CRT dissolution in FaSSIF. The excess enthalpy of CRT in different solvents was estimated by Chennuru et al. (2017). Remarkably, water has a negative excess enthalpy of −0.844 kcal/mol, supporting the formation of solvates and hydrates in the biorelevant dissolution.^51^

The dissolution of CRT is more effective in FeSSIF than in FaSSIF. The increase in the dissolution of CRT in FeSSIF for CAMs is approximately twofold higher than in the pure crystalline form. Moreover, the dissolution of NRG from CAMs exhibited a distinct enhancement, surpassing that of the pure NRG and physical mixtures. CRT:NRG 1:2 CAMs showed substantial improvement in all CAMs. A significant factor contributing to this augmented dissolution is micellar solubilization, which is crucial in improving CRT’s ability to dissolve. Bile salts in solution increase the thermodynamic activity of CRT, which leads to this improvement. In a study conducted by Indulkar et al. (2018), it was observed that cilnidipine, a calcium channel blocker, exhibited a remarkable 40- to 30-fold higher solubility in both crystalline and amorphous solubility when bile salts were present compared to compendial buffer.^52^ An alternative rationale for this behavior could be that the high sodium taurocholate (NaTC) concentration in FeSSIF prevents crystallization or delays the nucleation induction time. Chen et al. (2015) investigated nucleation induction times for the supersaturated solutions of 11 different compounds when bile salts, specifically NaTC, were present. They observed that regardless of these compounds, physical and chemical characteristics, the presence of NaTC extended the nucleation induction times.^53^ Nonetheless, the dissolution performance of CRT was improved in the case of CRT–NRG CAMs compared with pure crystalline CRT and NRG.

### 3.5 Physical Stability

Amorphous solids have a tendency to transform into more stable crystalline forms over time, so it is crucial for coamorphous systems (CAMs) to maintain their physical stability in the long term. In the case of CRT:NRG CAMs, they were subjected to storage at 40 °C for 180 days to investigate their potential crystallization. Interestingly, regardless of the molar ratio, all CAMs displayed diffraction patterns, showing no signs of crystallinity. This indicated that they retained a uniform, amorphous structure without any phase separation, as evident in Figure 9. The stability of CRT:NRG CAMs can be attributed to their relatively high glass transition temperature (*T*_g_), which exceeds 50 °C of the storage temperature. This higher *T*_g_ limits the molecular mobility of CAMs due to suppressed β relaxations at the storage temperature. This phenomenon is often referred to as the “*T*_g_-50K rule” or the Kauzmann temperature (*T*_k_), contributing to the physical stability of CAMs.^54,55^ Moreover, the strong intermolecular interactions observed in the CRT:NRG CAM’s FTIR spectra and the positive deviation of experimental *T*_g_ values from theoretical *T*_g_ values play significant roles in ensuring the physical stability of CAMs.^56^ In summary, the stability of CAMs is a multifaceted outcome resulting from both their high *T*_g_ concentration and the presence of strong intermolecular interactions.

**Figure 10:**
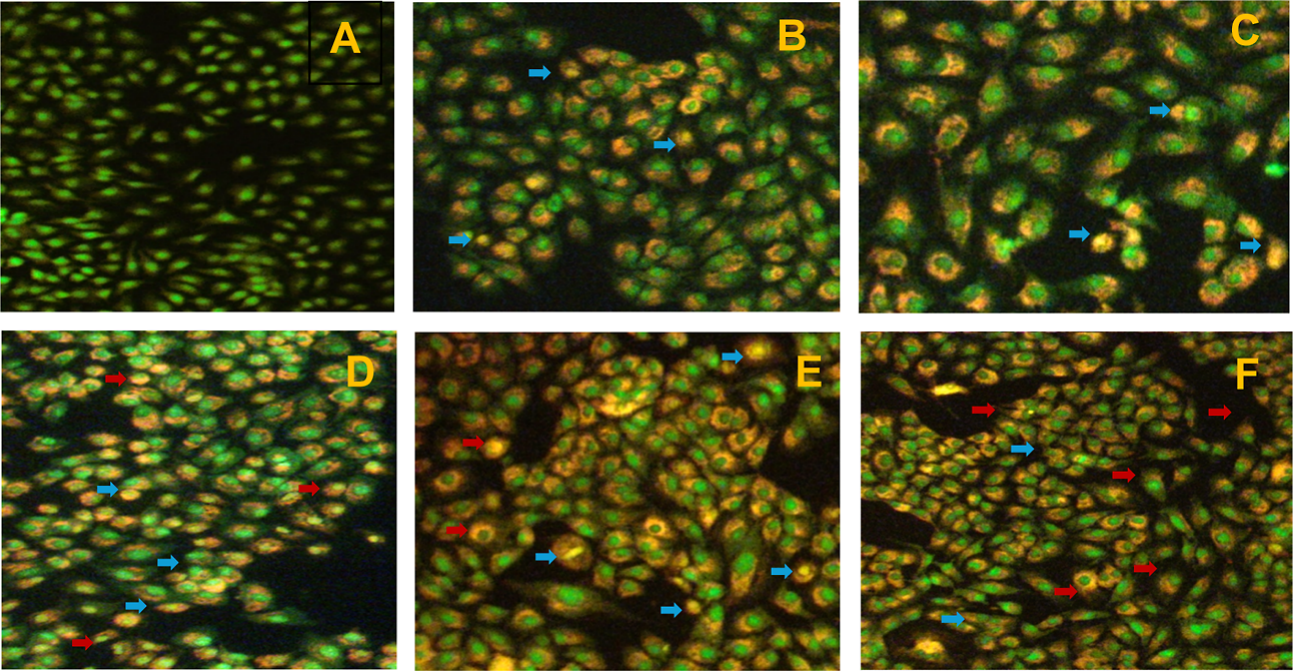
Apoptosis and necrosis detection using the AO/EB staining method. (A) Normal control, (B) CRT, (C) CNPM, and (D–F) CRT + NRG (1:1, 1:2, and 2:1) combination. Live cells (green), necrotic cells (blue arrows), and apoptotic cells (red arrows).

### 3.6 In Vitro Cell Line Studies

#### 3.6.1 AO/EB Staining

A549 cells were stained with AO/EB. While it is well known that AO imparts fluorescent green when bound to DNA by penetrating through all cell membranes, on the other hand, EB can only enter cells through broken membranes and bind to concentrated DNA fragments or apoptotic bodies, which appear as orange–red fluorescence. Furthermore, EB has a fluorescence intensity higher than that of AO. This approach distinguishes between normal, early, late, apoptotic, and necrotic cells. A549 cells were treated with IC_50_ concentrations of CRT, NRG, CNPM, and co-amorphous systems of CRT–NRG for 24 h, and the results are depicted in Figure 10. The AO/EB staining results demonstrated that CAMs have more morphologic traits of apoptotic A549 cells than the pure forms. Interestingly, the apoptotic cell percentage has substantially enhanced in CAMs of CRT–NRG compared to the normal, CRT, and NRG alone.

**Figure 11:**
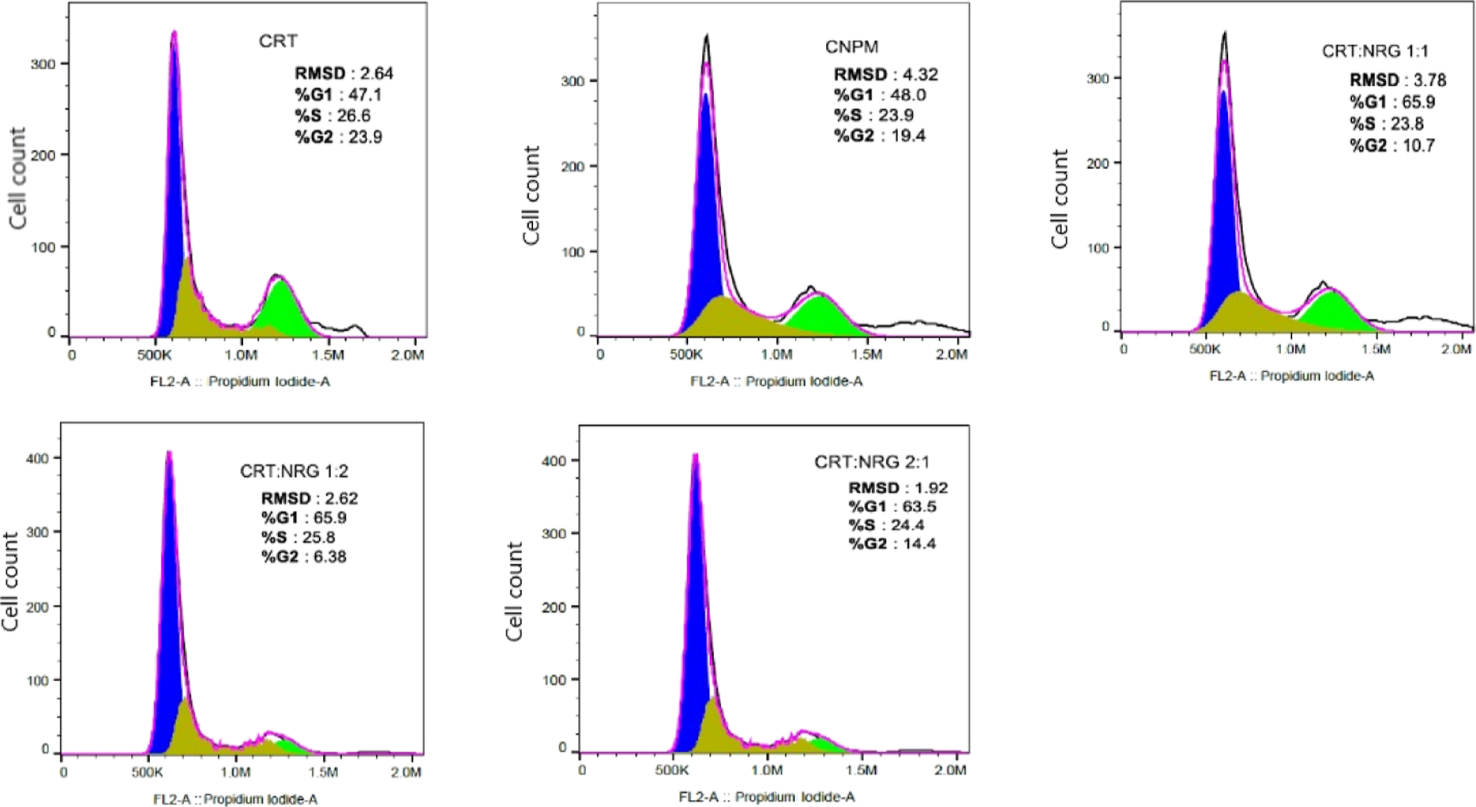
Cell cycle analysis of CRT, CNPM, and CAMs in A549 cells *in vitro* using flow cytometry.

#### 3.6.2 SRB Assay

The SRB assay was performed to calculate the percentage of cell death and the IC_50_ values of the treatment groups. The cytotoxicity test was carried out in a dose-dependent fashion. CRT, NRG, and CRT + NRG IC_50_ values were calculated to be 3530 nM, 162.30 μm, 3330 nM (CRT:NRG 1:1), 3340 nM (CRT:NRG 1:2), and 3090 nM (CRT/NRG 2:1), respectively. When compared to individual medication treatments, the treatment group taking the pharmacological combination CRT + NRG had a lower IC_50_ value (Table 2).

**Table 2:** SRB Experimental Results of CRT, NRG, and Coamorphous Systems

s.no.	sample	IC_50_ value (nM)
1	CRT	3530
2	NRG	162.30 (μm)
3	CRT:NRG 1:1	3330
4	CRT:NRG 1:2	3340
5	CRT:NRG 2:1	3090

#### 3.6.3 Cell Cycle Analysis

A549 cells were treated with CRT, NRG, CNPM, and CAMs (1:1, 1:2, and 2:1) of individual IC_50_ concentrations for 48 h. Cells were washed, fixed, and stained with propidium iodide after treatment, and the cell cycle status was determined using flow cytometry. The percentage of cells in the G0/G1 phase increased significantly for CAMs compared to pure CRT and CNPM (Figure 11). In the case of CRT and CRPM, the percentage of cells in the G0/G1 phase was 47.1 and 48.0, whereas for CAMs, it was 65.9, 65.9, and 63.5 (1:1, 1:2, and 2:1). Interestingly, the G2/M phase has reduced in CAMs 10.7, 6.3, and 14.4 (1:1, 1:2, and 2:1) than the pure CRT (23.9) and CNPM (19.4). Nevertheless, CAMs exhibited proapoptotic effects on A549 cell lines compared to pure CRT.

**Figure 12:**
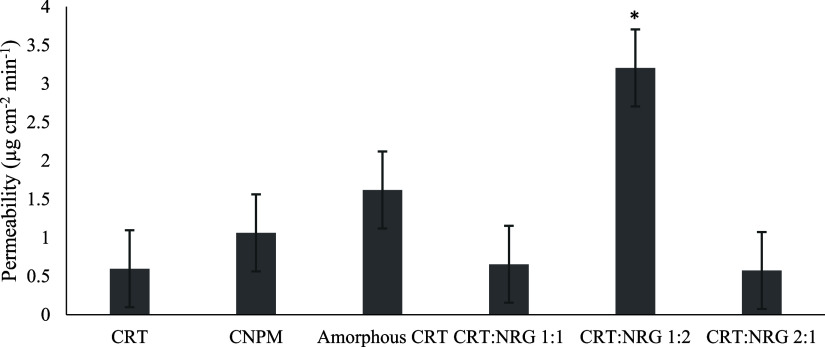
Permeability of CRT, CNPM, amorphous CRT, and CAMs. * implies a substantial difference when compared to CNPM (control). *p* < 0.05 significantly different.

The *in vitro* results showed that CAMs decreased A549 cell proliferation and arrested the cell cycle in the G0/G1 phase, indicating a reduced level of cell migration. The rationale for the improved apoptosis and reduced cell migration effect of CAMs than individual compounds could be attributed to the diminished expression of MMP-2 and MMP-9 proteins in A549 cells. Shi et al. (2020) reported a similar observation with naringenin, where cell migration was decreased for A549 lung cancer cells due to the reduction in the expression of MMP-2 and MMP-9 proteins.^57^ Aroui et al. (2015) studied the influence of NRG on glioma cells (U251) and distinguished the inhibition of NRG on the invasion and migration of cells. Furthermore, the molecular mechanism for inhibition activity was investigated at various concentrations of NRG, revealing the MMP-2 and MMP-9 downregulation and the inactivation of the p38 signaling pathway.^58^ Apart from these mechanisms, NRG showed apoptosis by inhibiting signaling pathways like PI3K/AKT/mTOR,^59^ EGFR via ganglioside buildup in GM3,^60^ activation of p38 mitogen-activated protein kinase, and caspase-3 protein in a dose-dependent fashion.^61^ Xu et al. (2021) studied the therapeutic effect of NRG and the fundamental mechanisms responsible for apoptosis and cell cycle analysis using Hoechst 33258 staining and flow cytometry in SNU-1 gastric carcinoma cells. The apoptosis and cell cycle results showed that NRG significantly induced apoptosis by blocking the PI3K/AKT pathway and the arrest of SNU-1 cells in the G0/G1 phase.^62^ These findings corroborate that CRT and NRG effectively inhibited human lung cancer cell proliferation, migration, and metastasis in *in vitro* studies compared to individual counterparts.

### 3.7 Permeability Studies (*Ex Vivo*)

A desirable and essential characteristic for increasing the bioavailability of CRT–NRG CAMs is permeability. CRT, CNPM, and CAMs permeability profiles are shown in Figure 12. The permeability of CRT was improved for CRT–NRG CAMs with respect to the physical mixture and individual compounds. CRT–NRG 1:2 CAMs exhibited the highest permeability, preceding the CRT–NRG 1:1 and CRT–NRG 2:1 molar ratios. However, only CRT–NRG CAMs 1:2 showed a statistically significant improvement in permeability, which is fivefold compared to pure CRT, while NRG showed statistically significant improvement for all CAMs. CRT–NRG 1:2 showed the highest permeability (fivefold) for NRG among all ratios of CAMs.

**Figure 13:**
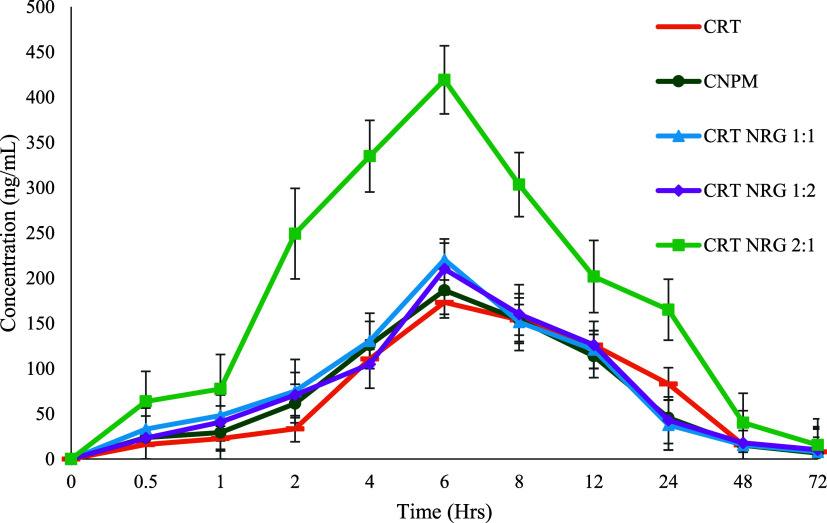
Pharmacokinetic profiles of CRT, CNPM, and CAMs (CRT:NRG 2:1 is only significantly different from the physical mixture). *n* = 3, *p* < 0.05, AUC_0–*t*_ from CAMs.

The enhanced permeability of CNPM to CRT can be correlated to the presence of NRG, which inhibits the P-gp transporter (efflux). Another hypothesis is *in situ* amorphization, particularly in light of the physical mixture of CRT–NRG contending with CAMs for permeability enhancement due to improved dissolution (Figure 8). This behavior was observed in the case of IND CAMs prepared with amino acids as coformers, succeeding in improved solubility.^63,64^ Furthermore, the significantly higher solubility of amorphous CRT relative to its crystalline form may account for the increase in the permeability of the former. The reduced drug release of CRT from the CAMs may be the factor responsible for the decrease in the permeability of 1:1 and 2:1 CRT:NRG CAMs. CRT:NRG 1:1 and CRT:NRG 2:1 showed analogous drug release to pure CRT in FaSSIF dissolution (Figure 8A). Interestingly, the extent of NRG dissolution positively correlates to CRT permeability from CAMs. This pattern enables us to anticipate the NRG ability to inhibit P-gp in a concentration-dependent fashion.^65^ When comparing NRG concentration among different ratios of compounds, it was observed that CRT:NRG 1:2 exhibited the highest, followed by CRT:NRG 1:1, CNPM, and CRT:NRG 2:1. Additionally, all concentrations of the compounds were above the IC_50_ value of NRG (2.4 mM) for P-gp inhibition. In light of this, the CRT:NRG 1:2 CAMs exhibited ameliorated permeability more than the other CAMs owing to a higher NRG concentration. The addition of NRG as a co-former to CRT resulted in enhanced solubility and inhibition of P-gp in CAMs of CRT, consequently leading to improved permeability compared to pure CRT.

### 3.8 *In Vivo* Oral Bioavailability Study

Assessing the effectiveness of co-amorphous formulations requires successfully translating the solubility advantage into *in vivo* bioavailability. As a control, a physical mixture (CNPM) of CRT and NRG was employed to distinguish the pharmacokinetic and formulation parameters. CRT plasma concentration was determined in male Wistar rats for 72 h at 15 mg/kg, with a dose of CRT, CRT-NRG physical mixture, and an equivalent amount of CRT-NRG CAMs (1:1, 1:2, and 2:1 ratios).

A maximum plasma concentration (*C*_max_) of 173 ng/mL was observed with CRT after 6 h. The prepared CAMs showed higher *C*_max_ than pure CRT, regardless of the molar ratio. Specifically, compared to pure crystalline CRT, the CRT:NRG 2:1 increased the *C*_max_ by 2.4-fold, followed by the CRT:NRG 1:1 and CRT:NRG 1:2 by 1.2-fold (Figure 13).

Likewise, the enhancement in the extent of absorption (AUC) followed the same pattern in CAMs. CRT:NRG 2:1 showed the most significant increase in AUC (2.1-fold) compared to pure CRT, as indicated in Table 3, surpassing all other CAMs. For statistical analysis among groups, one-way ANOVA (Dunnett’s test) was performed using GraphPad Prism version 8.0. PumasCP software was used for the analysis of pharmacokinetic parameters employing a noncompartmental model. A statistically significant difference (*p*-value 0.05) was observed in CRT:NRG 2:1 CAMs compared with other CAMs. The rapid absorption of the drug in the GIT is the factor that causes the increase in CRT’s bioavailability. It is plausible that the enhanced solubility of CRT in CAMs contributed to its faster dissolution rate in the GIT and quick assimilation into the systemic circulation. This enhanced solubility could have played a role in expediting CRT’s transit. Furthermore, it was postulated that the coexistence of NRG and CRT in the luminal fluid was essential for saturating the P-gp transporters, which may have increased the permeability and absorption of CRT.^38,66^

**Table 3:** Pharmacokinetic Characteristics Following a Single Oral Dose (15 mg/kg) of CRT, CNPM, and CAMs (*n* = 3)

s.no.	parameter	CRT	CNPM	CRT/NRG 1:1	CRT/NRG 1:2	CRT/NRG 2:1
1	AUC_0–*t*_ (ng h/mL)	3584.63 ± 217.65	3830.92 ± 870.41	4699.30 ± 1086.10	4309.61 ± 488.75	8793.96 ± 171.08^a^
2	*C*_max_ (ng/mL)	173.26 ± 5.34	186.55 ± 30.67	220.62 ± 115.34	210.12 ± 14.90	419.28 ± 18.13^a^
3	*T*_max_ (h)	6	6	6	6	6
4	*t*_1/2_ (h)	22.13	26.88	18.40	20.15	17.54
5	MRT (h)	20.19 ± 0.75	19.25 ± 1.34	18.85 ± 5.99	19.77 ± 1.93	21.35 ± 1.18

a A substantial difference when compared to the control (CNPM). *p* < 0.05 significantly different.

It is interesting to notice that CRT functions as CYP3A4’s substrate and inhibitor. In spite of having a lower NRG content than other formulations, the CRT:NRG 2:1 formulation was found to have the maximum bioavailability in the current investigation. The maximum bioavailability was attained with CRT:NRG 2:1, suggesting a positive correlation between CRT concentration and the inhibition of CYP3A4 by CRT. Hurtado et al. (2021) used midazolam and s-warfarin as reference compounds to examine the effects of CRT on concurrently administered medications metabolized by CYP3A and CYP2C9. Thirty-three adult NSCLC patients participated in this study to investigate possible drug interactions. Only the AUC of S-warfarin was improved by (56%), whereas CRT considerably enhanced the *C*_max_ and AUC of midazolam (by 1.82- and 5.42-fold, respectively), when compared to midazolam alone.^21^ This suggests that CYP3A4 is inhibited in a dual fashion by CRT (autoinhibition) and NRG, which may contribute to the drug’s rapid absorption in the GIT. These findings highlight the importance of considering potential interactions with concomitant medications when prescribing along with CRT. The CAMs of CRT:NRG enhanced the permeability and bioavailability of CRT regardless of their molar ratio.

## 4 Conclusions

The current study employed computational modeling to develop CAMs of CRT with enhanced solubility and bioavailability. The simulation results for the docking score and binding energy were used to determine the ideal coformer combination for CRT. The solvent evaporation process was used to prepare CAMs with NRG as a co-former. CRT-NRG CAM’s phase solubility and miscibility were confirmed by measuring the solubility parameters and identifying a single *T*_g_. The presence of specific chemical interactions between the CRT and NRG was suggested by the significant divergence between the estimated and observed *T*_g_ values by the Gordon–Taylor equation, which FTIR corroborated. Furthermore, CRT-NRG CAMs increased CRT solubility and permeability compared with crystalline CRT and CNPM. CAMs decreased the percentage viability of cells in *in vitro* cytotoxicity experiments. Compared to the negative control, CAMs suspended the G0/G1 phase of the cell cycle and prevented cell growth in the A549 cells. In light of improved solubility and NRG’s dose-dependent inhibition of P-gp, CRT’s permeability was enhanced. In conclusion, studies conducted on male Wistar rats showed that CRT-NRG CAMs significantly improved the CRT bioavailability.

## Supporting Information Available

Chemical structure of CRT, chemical structure of NRG, 3D interaction diagram and position of CRT and NRG, experimental and theoretical glass transition temperature, pi–pi interactions, chemical structure and docking score of CRT and NRG, and 2D interaction diagram (PDF)

## Acronyms

CAMs: coamorphous materials
CRT: ceritinib
NRG: naringin
NSCLC: nonsmall cell lung cancer
SCLC: small cell lung cancer
GIT: gastrointestinal tract
IFD: induced fit docking
GRAS: generally regarded as safe
MD: molecular dynamics
CNPM: physical mixture of CRT–NRG
CRT–NRG CAMs: coamorphous materials of ceritinib and naringin
CRT–NRG 1:1: coamorphous materials of ceritinib and naringin of 1:1 molar ratio
CRT–NRG 1:2: coamorphous materials of ceritinib and naringin of 1:2 molar ratio
CRT–NRG 2:1: coamorphous materials of ceritinib and naringin of 2:1 molar ratio
HPLC: high-performance liquid chromatography
HPMC: hydroxypropyl methylcellulose
FaSSIF: fasted-state simulated intestinal fluid
FeSSIF: fed-state simulated intestinal fluid
SRB: sulforhodamine B
AO/EB: acridine orange/ethidium bromide
PBS: phosphate-buffered saline
CPCSEA: Control and Supervision of Experiments on Animals
MAHE: Manipal Academy of Higher Education
IAEC: Institutional Animal Ethics Committee
RMSD: root-mean-square deviation
RMSF: root-mean-square fluctuation
NaTC: sodium taurocholate
