# Supplementary material for: Computational-Based Polyphenol Therapy for Nonsmall Cell Lung Cancer: Naringin Coamorphous Systems for Solubility and Bioavailability Enhancement
Source: Mol Pharm. 2024 Jul 25;21(8):3951–66. doi: 10.1021/acs.molpharmaceut.4c00303 (PMC11304383; doi:10.1021/acs.molpharmaceut.4c00303)
Supplement: Supplementary file 1 — mp4c00303_si_001.pdf [file mp4c00303_si_001.pdf]

A computational-based Polyphenol therapy for Non-Small Cell Lung Cancer: Naringin co-amorphous systems for solubility and bioavailability enhancement.

*Dani Lakshman Yarlagadda [a], Subham Das [b], Vullendula Sai Krishna Anand [a], Suman Manandhar [c], Swapnil J Dengale [d], K Sreedhara Ranganath Pai [c], Krishnamurthy Bhat [a]**

a. Department of Pharmaceutical Quality Assurance, Manipal College of Pharmaceutical Sciences, Manipal Academy of Higher Education (MAHE), Manipal, Karnataka – 576104, India.
b. Department of Pharmaceutical Chemistry, Manipal College of Pharmaceutical Sciences, Manipal Academy of Higher Education (MAHE), Manipal, Karnataka – 576104, India.
c. Department of Pharmacology, Manipal College of Pharmaceutical Sciences, Manipal Academy of Higher Education (MAHE), Manipal 576104, India.
d. Department of Pharmaceutical Analysis, National Institute of Pharmaceutical Education and Research (NIPER), Guwahati, Changsari 781101, India

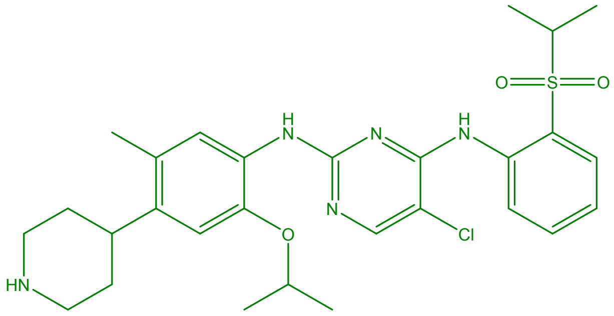

Figure S1A. Chemical Structure of Ceritinib

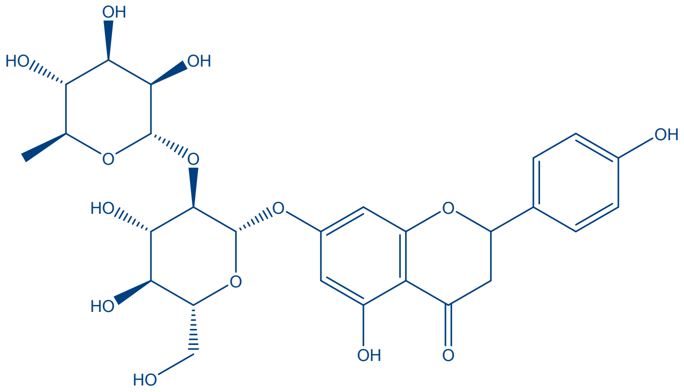

Figure S1B. Chemical Structure of Naringin

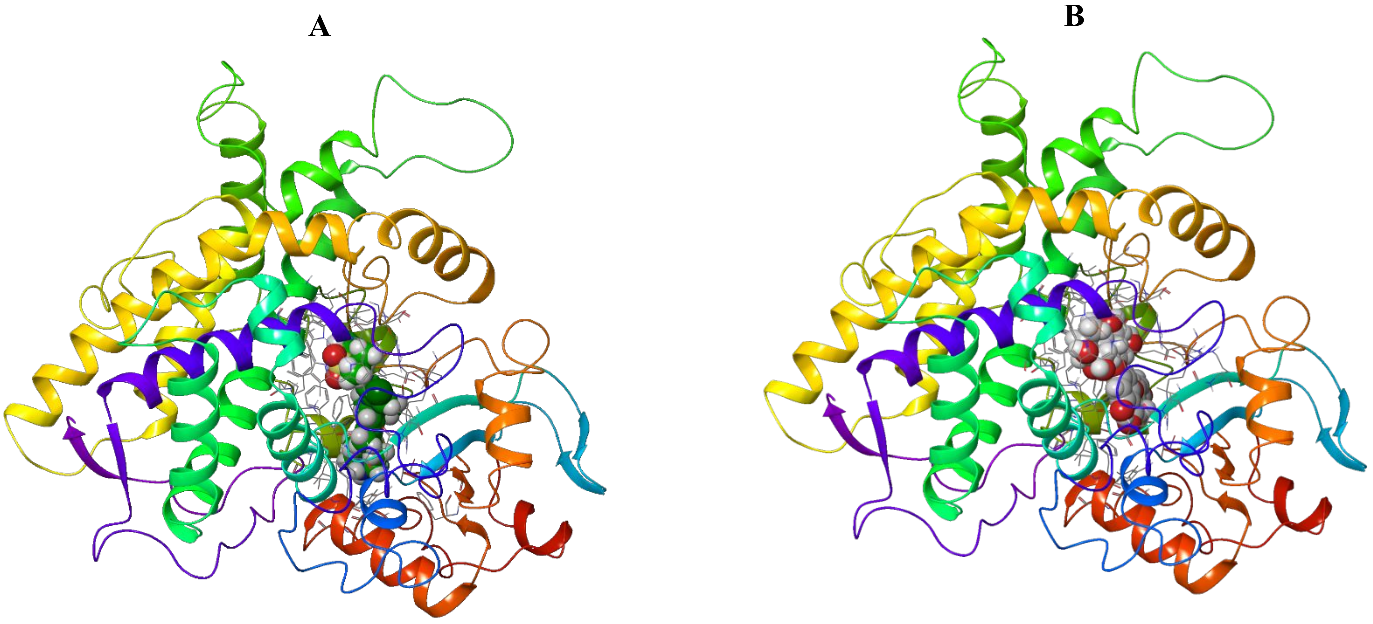

Figure S2. 3D interaction diagram and position of CRT and NRG in the molecular surface of the CYP3A4 protein. (CRT and NRG are highlighted in animated cartoon shapes for better understanding).

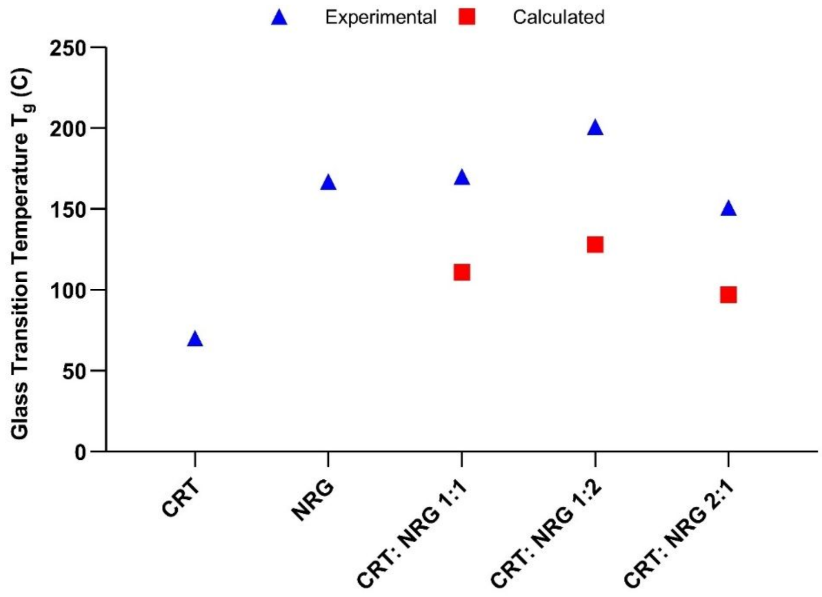

Figure S3. Experimental and Theoretical glass transition temperature (Tg) of Ceritinib (CRT), Naringin (NRG), and co-amorphous systems using Gordon Taylor equation.

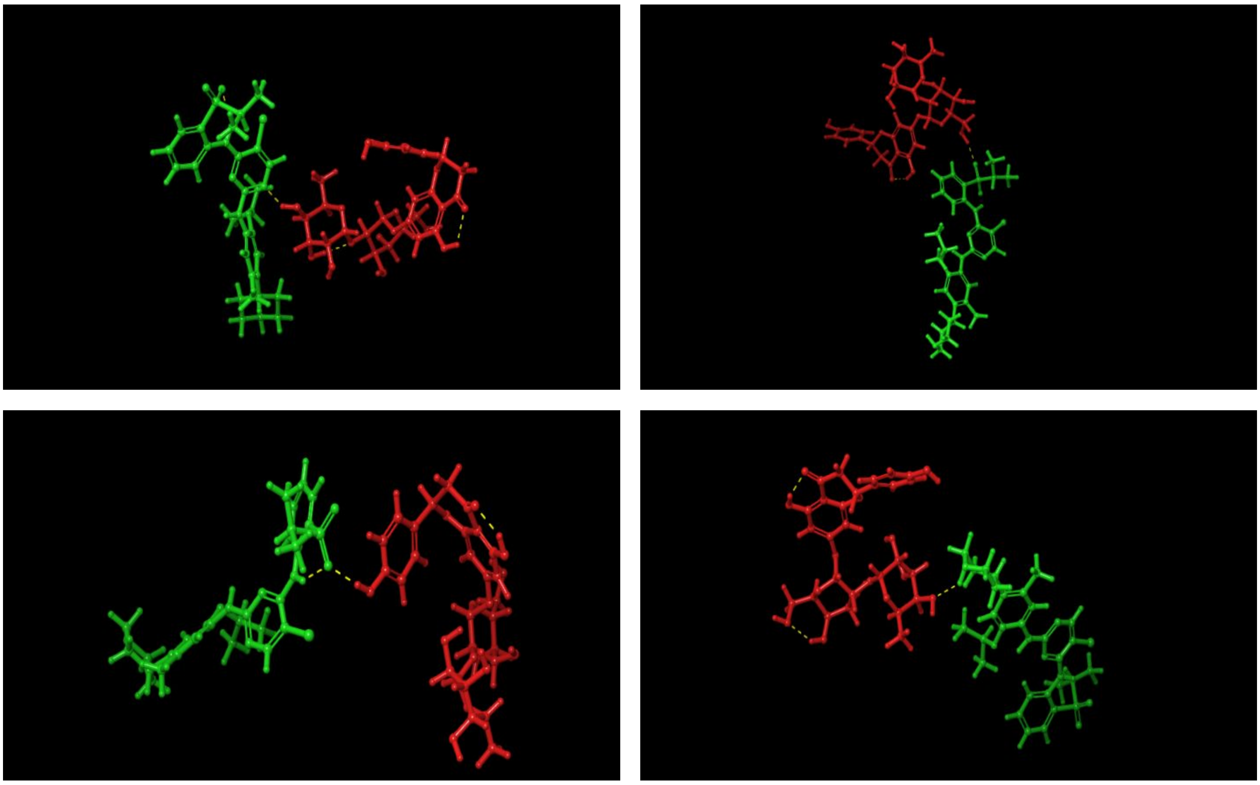

Figure S4. pi-pi interactions between CRT-RTH systems from MD simulation. (Red-CRT, Green- RTH, Blue- pi-pi interactions, Yellow- Hydrogen bonds)

Table S1. NRG and CRT, along with their chemical structure and docking score.

Compound name	Chemical structure	Docking score (Kcal/mol)
Naringin	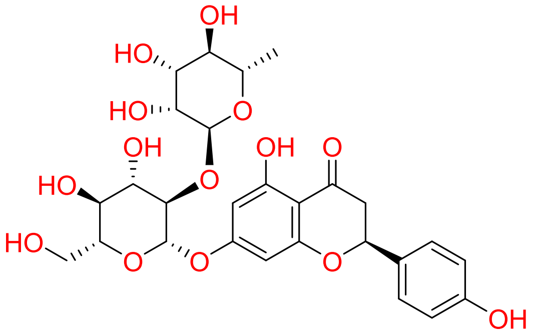	-10.970
Ceritinib	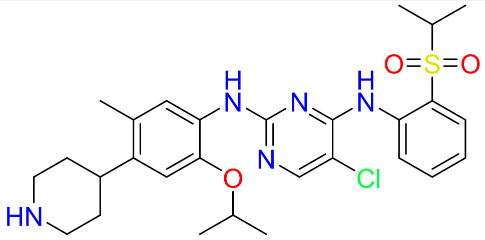	-9.035

Table S2. 2D interaction diagram alongwith binding interaction of NRG and CRT at the binding site of the CYP3A4 protein

Compound name	Chemical structure	Binding interactions
Naringin	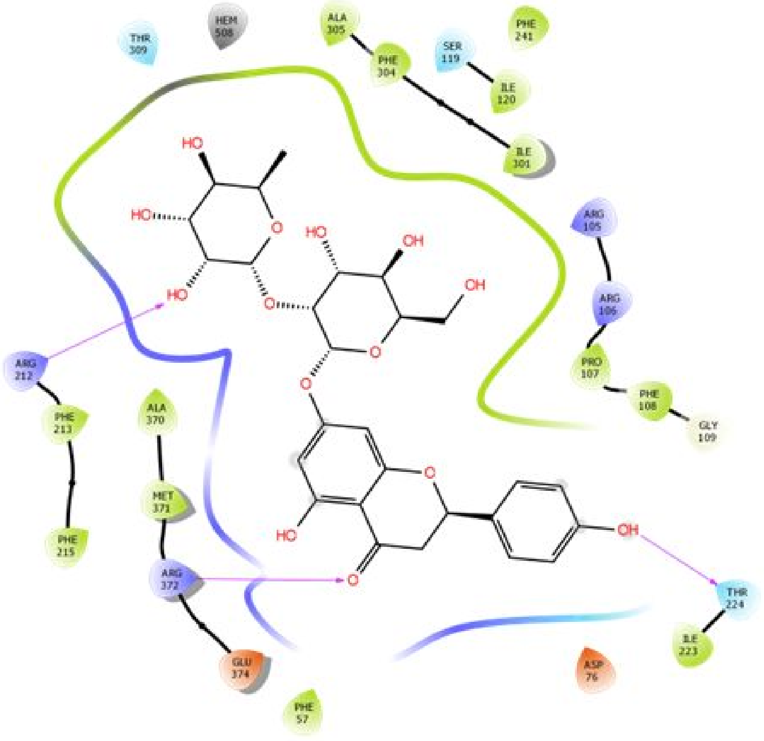	ARG212, THR224, ARG372 (H-bond interaction)

Ceritinib	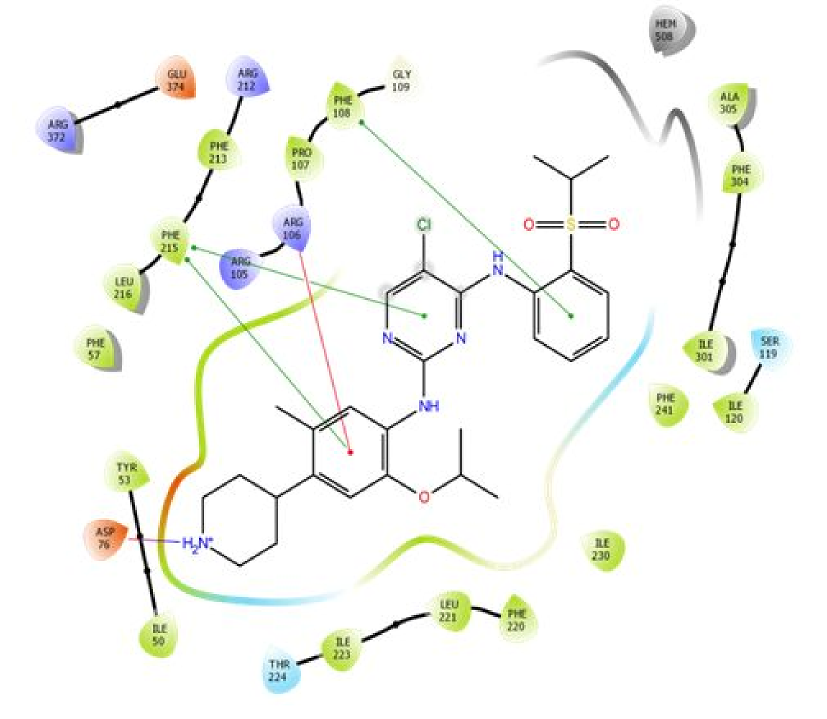	ASP76 (salt bridge), ARG106 (pi-cation), PHE108, PHE215 (pi-pi stacking)
